# A Phylogenetic Analysis of Chloroplast Genomes Elucidates the Relationships of the Six Economically Important *Brassica* Species Comprising the Triangle of U

**DOI:** 10.3389/fpls.2017.00111

**Published:** 2017-02-02

**Authors:** Peirong Li, Shujiang Zhang, Fei Li, Shifan Zhang, Hui Zhang, Xiaowu Wang, Rifei Sun, Guusje Bonnema, Theo J. A. Borm

**Affiliations:** ^1^Chinese Cabbage Department, Institute of Vegetables and Flowers, Chinese Academy of Agricultural SciencesBeijing, China; ^2^Plant Breeding, Wageningen University and ResearchWageningen, Netherlands

**Keywords:** chloroplast genome, assembly, *Brassica*, phylogeny, variation, divergence

## Abstract

The *Brassica* genus comprises many economically important worldwide cultivated crops. The well-established model of the *Brassica* genus, U’s triangle, consists of three basic diploid plant species (*Brassica rapa*, *Brassica oleracea*, and *Brassica nigra*) and three amphidiploid species (*Brassica napus*, *Brassica juncea*, and *Brassica carinata*) that arose through interspecific hybridizations. Despite being extensively studied because of its commercial relevance, several aspects of the origin of the *Brassica* species and the relationships within and among these six species still remain open questions. Here, we successfully *de novo* assembled 60 complete chloroplast genomes of *Brassica* genotypes of all six species. A complete map of the single nucleotide variants and insertions and deletions in the chloroplast genomes of different *Brassica* species was produced. The chloroplast genome consists of a Large and a Small Single Copy (LSC and SSC) region between two inverted repeats, and while these regions of chloroplast genomes have very different molecular evolutionary rates, phylogenetic analyses of different regions yielded no contradicting topologies and separated the *Brassica* genus into four clades. *B. carinata* and *B. juncea* share their chloroplast genome with one of their hybridization donors *B. nigra* and *B. rapa*, respectively, which fits the U model. *B. rapa*, surprisingly, shows evidence of two types of chloroplast genomes, with one type specific to some Italian broccoletto accessions. *B. napus* clearly has evidence for two independent hybridization events, as it contains either *B. rapa* chloroplast genomes. The divergence estimation suggests that *B. nigra* and *B. carinata* diverged from the main *Brassica* clade 13.7 million years ago (Mya), while *B. rapa* and *B. oleracea* diverged at 2.18 Mya. The use of the complete chloroplast DNA sequence not only provides insights into comparative genome analysis but also paves the way for a better understanding of the phylogenetic relationships within the *Brassica* genus.

## Introduction

Chloroplast genome sequences have been used extensively for inferring plant phylogenies for several reasons. Chloroplast genomes of plants are known to be highly conserved and have a smaller effective population size and thus shorter coalescent time when compared with nuclear genomes (Birky et al., 1983; Jansen et al., 2007). Whole chloroplast genome analysis is known to provide high resolution plant phylogenies (Parks et al., 2009). The chloroplast genomes of angiosperms can be divided into large single copy (LSC) and small single copy (SSC) regions separated by inverted repeat (IR) regions (Palmer, 1985). Substitution rates of chloroplast genomes are much lower than those of nuclear genomes, and are further significantly reduced in the IR regions of chloroplast genomes compared to their LSC and SSC regions (Wolfe et al., 1987). Despite this low rate of change, progress in inferring phylogenetic relationships at lower taxonomic levels has been made using chloroplast DNA sequences. Examples are use of chloroplast genomes to find the origin of domesticated citrus species (Carbonell-Caballero et al., 2015) and apple varieties (Nikiforova et al., 2013), and to elucidate the difficult phylogeny at lower taxonomic levels in intractable plant groups, such as the Arundinarieae tribe (Ma et al., 2014). The chloroplast genome sequence of *B. rapa* is a circular unit of 153,482 bp length, composed of a pair of IR regions (26,212 bp each), a LSC region (83,282 bp), and a SSC region (17,776 bp) (Wu et al., 2012).

The *Brassica* genus comprises many economically important crops cultivated worldwide. The species demonstrate extreme morphological diversity and crop forms; for example, *B. rapa* includes Chinese cabbage (*B. rapa* L. ssp. *pekinensis*), turnip (ssp. *rapa*), Pak-choi (*B.* ssp. *chinensis*) and oil types (ssp. *oleifera*), and *B. oleracea* includes among others cauliflower (*B. oleracea* L. var. *botrytis*), broccoli (var. *italica*) and cabbage (var. *capitata*). The phylogeny of these species and their subspecies is not fully understood (Song et al., 1988).

The *Brassica* genus belongs to the Brassicaceae family which is composed of 338 genera and 3,709 species (Warwick et al., 2006). *B. rapa* (AA), *B. nigra* (BB), and *B. oleracea* (CC) are three basic diploid species that gave rise to three amphidiploid species, *B. napus* (AACC), *B. juncea* (AABB), and *B. carinata* (BBCC) through the interspecific hybridizations *B. rapa* × *B. oleracea*, *B. rapa* × *B. nigra*, and *B. nigra* × *B. oleracea*, respectively, as modeled in U’s triangle (Nagaharu, 1935). Although most studies on the species in U’s triangle have confirmed the diploid origins of the amphidiploid species, many questions remain concerning how and when the amphidiploid species evolved from their parental diploids (Prakash and Hinata, 1980). Also, very little is known about the origins of the diploid species themselves. Crosses between *Brassica* species to introgress specific traits or to broaden the gene pool, have likely also affected the maternal genome composition of the resulting progenies (Palmer et al., 1983; Qiu et al., 2006). Therefore, the *Brassica* genus provides an opportunity to study the origin and evolution of species over a wide range of timescales.

Cultivated *Brassica* species and closely related *Arabidopsis* diverged from a common ancestor, however, estimates of divergence time are controversial. Several studies estimate the divergence date around 20 million years ago (Mya); 24 Mya based on nuclear sequence variation (Koch et al., 2000), 14.5–20.4 Mya based on mitochondrial genes (Yang et al., 1999) and 23.35 Mya (Hohmann et al., 2015) based on the chloroplast genome calibrated using fossil evidence. Yet another study that also uses chloroplast genome data calibrated using evidence from four different fossils estimates this event at 43 Mya (Beilstein et al., 2010). Consequently, estimates of the divergence of *B. rapa* and *B. oleracea* from *B. nigra* also range from 20 Mya (Arias et al., 2014), till 7.9 Mya (Lysak et al., 2005). Divergence time of *B. rapa* and *B. oleracea* based on diverse nuclear sequences was estimated 3.7–4 Mya (Inaba and Nishio, 2002; Liu et al., 2014) or 1.37–0.12 Mya (Cheung et al., 2009). *B. napus* was formed 7500–12500 years ago by interspecific hybridization between *B. rapa* and *B. oleracea* (Chalhoub et al., 2014). From the above it is evident that divergence estimates within the *Brassica* genus are controversial (Lysak et al., 2005; Cheung et al., 2009; Beilstein et al., 2010; Arias et al., 2014; Hohmann et al., 2015).

Based on chloroplast DNA (Warwick and Black, 1991) and mitochondrial DNA (Palmer and Herbon, 1988), the *Brassica* species are divided into two evolutionary lineages: the nigra lineage and the rapa/oleracea lineage. Several studies concluded that formation of *B. napus* via hybridization occurred multiple times involving different maternal genotypes. RFLP marker analysis of both chloroplast and mitochondrial DNA of 46 accessions representing *B. rapa*, *B. oleracea*, *B. montana*, and *B. napus* indicated four major cytoplasm types, representing almost all *B. rapa*, broccoletto (a *B. rapa* subspecies), *B. oleracea*, and *B. montana* (Song and Osborn, 1992). These authors also provided evidence for multiple origins of *B. napus* and showed identical chloroplast RFLP patterns between *B. montana* and *B. napus.* In another study, chloroplast SSRs and nuclear RFLPs were used to identify the ancestors of *B. napus* (Allender and King, 2010). This study also concluded that multiple hybridization events with different maternal genotypes resulted in *B. napus*, and suggested as the maternal donors both weedy *B. rapa* accessions and broccoletto accessions with divergent chloroplast genomes, but found no indication for *B. montana* as maternal parent. These studies are based on genetic markers (RFLP, AFLP, SSRs) that only characterize a small part of the genomes.

Although previous studies on *Brassica* phylogenetic relationships were based on nuclear DNA markers (Song et al., 1990; Allender and King, 2010) or on partial cytoplasmic DNA sequences (Palmer et al., 1983; Warwick and Black, 1997; Allender and King, 2010), the analysis of the complete chloroplast genome should render phylogenetic relationships with stronger support. The recent availability of nuclear (Wang et al., 2011; Chalhoub et al., 2014; Liu et al., 2014; Parkin et al., 2014) and chloroplast (Wu et al., 2012; Hao et al., 2014; Prabhudas et al., 2015) reference genomes of *Brassica* species enables such more detailed studies of the origin and phylogenetic relationships within the Brassica’s. We assembled 60 *de novo* chloroplast genomes representative of the economically important *Brassica* species and constructed a maternal phylogeny. The study of the distribution of mutational events, such as single nucleotide variants (SNVs), deletions and insertions along the evolutionary history of the *Brassica* genus, allowed inference of the origin of the amphidiploid species. Additionally, this comparative analysis also produced new insights into divergence times between *Brassica* species.

## Materials and Methods

### Plant Material and DNA Extraction

Plant materials sequenced in this study were provided by the Chinese Cabbage Research Group of the Institute of Vegetables and Flowers, Chinese Academy of Agricultural Sciences (CAAS-IVF), from the Dutch Crop Genetic Resources Center (CGN) in Wageningen UR and from Bejo and Rijk Zwaan breeding companies, comprising a total of 60 *Brassica* accessions (**Supplementary Table S1**). For the accessions that were inbred or DH lines, DNA was isolated from 50 to 100 two-week-old seedlings (hypocotyls and cotyledons) or from leaves from a single plant. For GeneBank accessions, DNA was isolated from single plants, as accessions are generally heterogeneous. In the paper, we use the term accession for both the single plants representing GeneBank accessions and the DH or inbred lines from breeding companies. Total DNA was extracted from seedlings (hypocotyls and cotyledons) or fresh leaves of the 60 *Brassica* accessions by the conventional cetyltrimethylammonium bromide (CTAB) method (Chen and Ronald, 1999) and stored at -80°C. Chloroplast genome sequences of *A. thaliana* (NC_000932), *A. lyrata* (LN877383), *B. rapa* (DQ231548), *B. juncea* (KT581449), *B. oleracea* (KR233156), *B. nigra* (KT878383) and *B. napus* (KP161617, GQ861354, KM454973 and KJ872515) were obtained from the original publication.

### Genome Sequencing

Whole genome sequences were generated using Illumina HiSeq2000 and HiSeq3000. In brief, short-insert (400-bp) libraries were constructed using the TruSeq^TM^ DNA sample preparation kit (Illumina, USA) for Illumina HiSeq sequencing and following the manufacturer’s protocol (Illumina). DNA from the different samples was indexed by tags and pooled together in one lane of Illumina’s Genome Analyzer for sequencing. Raw reads were first filtered to obtain the high-quality clean data by removing adaptor sequences and low-quality reads with Q-value ≤20.

### Chloroplast Genome Assembly, Annotation, Alignment, and Visualization

The chloroplast genomes were assembled using a highly automated five stage pipeline (manuscript in preparation). Briefly this pipeline consists of the following stages: (1) Extraction of a k-mer frequency table from raw data and detection of two linked peaks at frequency N and 2^∗^N, representing single copy and IR regions of the chloroplast genome, respectively, and extraction of underlying k-mers. (2) Extraction of reads containing these k-mers and initial assembly using a parameter spread. (3) Refinement of initial assembly by iterative selection of additional reads from raw data based on previous assembly and subsequent reassembly. (4) Local selection and reassembly of additional reads near scaffold ends to alleviate problems caused by variable representation of the chloroplast genome in the sequencing library. (5) Gap filling by local selection and *de novo* assembly of these gap-filling reads and insertion of gap-filling contigs into the scaffolds. This pipeline takes into account the circular and quadripartite [(LSC, IR (twice) and SSC] nature of the chloroplast genome, and may, if data supports this, produce finished circular chloroplast genome assemblies. The assembled chloroplast genomes were deposited to BRAD^1^.

The chloroplast genes were annotated using an online DOGMA tool (Wyman et al., 2004), using default parameters to predict protein-coding genes, transfer RNA (tRNA) genes, and ribosome RNA (rRNA) genes. Start and stop codons of protein-coding genes were searched and determined by BLASTX against the NCBI protein database, with *B. rapa* as a guide. Genome maps were drawn with OGDraw (Lohse et al., 2007). Multiple sequence alignments were performed in MAFFT version 5 (Katoh et al., 2005) under standard parameters, and adjusted manually where necessary. Full alignments with annotations were visualized using the VISTA viewer (Frazer et al., 2004). Pairwise assembly alignments were made between the reference genome and the *de novo* assembled genomes using MUMmer (Kurtz et al., 2004).

### Variant Calling

Sample sequences were mapped against the *B. rapa* chloroplast genome (Chiifu). The mapping procedure applied in this study is summarized as follows: Burrows–Wheeler Aligner (BWA 0.6.2) software was used to map paired-end reads in this study (Li and Durbin, 2010), and reads with low-quality alignments were then filtered out from mapping using SAMtools (Li et al., 2009) with the default parameters. The final set of mapping files (BAM) was used to perform a multisample variant calling by using BCFtools. The calling parameters were adjusted to obtain both SNVs and InDels.

### Phylogenetic Analysis

To evaluate consistency of phylogenetic trees produced from regions with different molecular evolutionary rates (Palmer, 1985), we extracted three subsets (LSC, SSC, and IRs) from the complete chloroplast data set, and combined these to produce three types of trees.

The program JModeltest 2 was used to find the optimal substitution model for each subset (Posada and Crandall, 1998; Darriba et al., 2012), using both the Bayesian information criterion (Felsenstein, 1988) and the Akaike information criterion (Akaike, 1974). Models for three datasets were explored: LSC+SSC dataset (GTR+G model), IR dataset (GTR+I model) and whole chloroplast genome (GTR+I+G model). Assembled chloroplast sequences were aligned with the help of MAFFT software (Katoh et al., 2005). The maximum likelihood (ML) tree was computed in PhyML (Guindon et al., 2010) using settings of the best-fitting model tested by JModeltest program (Darriba et al., 2012). The method used was a maximum-likelihood iterative model and a bootstrap of 1,000 repetitions was used to assess the reliability of the phylogeny reconstructed.

### Estimation of Divergence Times within *Brassica*

Divergence times of *Brassica* species were estimated using the BEAST program with the Bayesian method (Drummond et al., 2012). Uncorrelated relaxed lognormal clock was used in our analysis that allows different rates to be optimized independently on each branch of the tree. The Yule process described the likelihood of speciation for this analysis (Yule, 1925), and branching rates were determined under an optimal GTR+I+G nucleotide model. Two independent Markov chain Monte Carlo runs in BEAST were conducted for 500,000,000 generations sampling every 50,000 generations for the chloroplast genome analysis. To calibrate the phylogenetic tree of the chloroplast genomes of the accessions belonging to the six *Brassica* species, estimations of dates of speciation events calculated by valid fossil evidence were used (Hohmann et al., 2015). In our experiment, *Arabidopsis* was the outgroup, and the root age was constrained by a normal distribution with a mean of 23.35 Mya. In addition, the divergence age of *A. thaliana* and *A. lyrata* was set at 5.97 Mya. We then used Tracer v. 1.6 to explore the output of BEAST (Rambaut et al., 2014). TreeAnnotator v. 1.8 (part of the BEAST package) was used to produce maximum clade credibility trees from the postburn-in trees and to determine the 95% posterior density of ages for all nodes in the tree.

## Results

### Characteristics of Data Sets and Chloroplast Assembly

Sixty sequenced chloroplast genomes of *Brassica* genotypes together with seven published chloroplast genomes were used in the present study, including chloroplast genomes of 30 *B. rapa* accessions representing nine different morphotypes, of 17 *B. oleracea* accessions representing seven different morphotypes, of eight *B. napus* accessions representing oilseed and swede morphotypes, of eight *B. juncea* accessions representing five morphotypes, of one *B. nigra* and of one *B. carinata*; as outgroup one chloroplast genome each of *A. thaliana* and *A. lyrata* was included (**Supplementary Table S1**). The raw reads were mapped onto the *B. rapa* subsp. *pekinensis* (Chiifu, DQ231548.1) chloroplast genome as described in the Section “Materials and Methods.” Mapped reads of each genotype for the sequences of 60 whole chloroplast genomes are listed in **Supplementary Table S1**. The chloroplast genome coverage of each sample was very high (**Supplementary Table S1**) because of the numerous copies of chloroplasts in leaf cells (Zhang et al., 2011).

**FIGURE 1:**
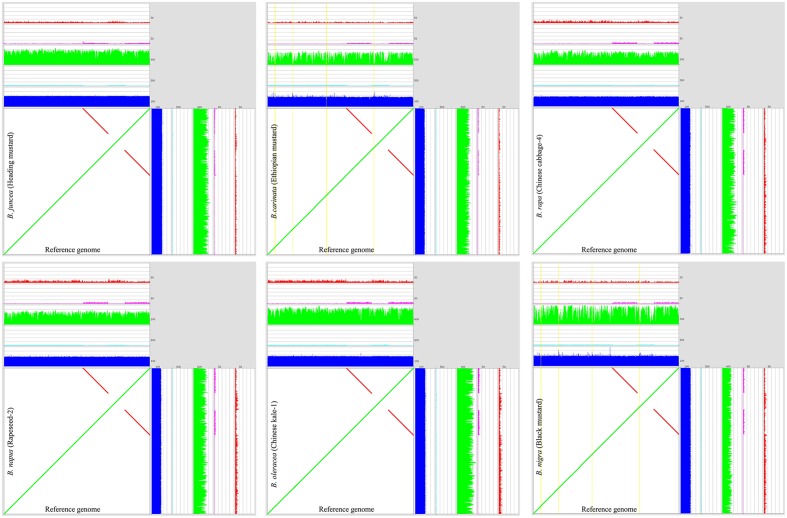
**Comparison of the *de novo* assembled chloroplast genomes (along the *Y*-axis) with the reference chloroplast genome of *B. rapa* Chiifu (along the *X*-axis) visualized in a mummer-plot.** In the MUMmer-plots, yellow lines denote areas with paired-end coverage of less than 10% of the average. The colored tracks shown in each figure are the average fragment length of the paired-end data (blue), relative number of non-mapped base pairs (cyan), paired-end sequenced DNA fragments coverage (green), nucleotide coverage by discordant read-pairs (pink) and nucleotide coverage by linking read-pairs (red), respectively.

In this study, we used k-mer frequency histograms to identify and extract the chloroplast reads from total DNA sequencing data (**Supplementary Figure S1**) and assembled chloroplast genomes for these accessions using a highly integrated and automated pipeline. Dot plot analyses using MUMmer software was performed to compare *de novo* assembled genomes with the reference genome (**Figure 1**). Assembled genomes of all six *Brassica* species are collinear to previously published chloroplast genomes of *Brassica* (Wu et al., 2012; Hao et al., 2014), as no rearrangements were identified. Their genome sizes are very similar, ranging from 152860 to 153700 bp (**Supplementary Table S1**).

### Variant Analysis in Chloroplast Genomes of the *Brassica* Genus

A total of 2646 SNVs and 464 insertions and deletions (InDels) were detected by Burrows–Wheeler Aligner software (BWA) and SAMtools, summing up to a total of 3110 high-quality variant positions (**Supplementary Tables S2** and **S3**). Manual curation and the high coverage used in this study allowed the detection of discrepancies with the reference genome that can most probably be attributed to Sanger sequencing errors in the original sequence. For example, position 116457 (A) of the *B. rapa* subsp. *pekinensis* (Chiifu) chloroplast genome that we re-sequenced was different from the reference (G, DQ231548.1). The number of changes observed for all the four nucleotides among the 2646 SNVs were 52.4% A or T, 47.6% G or C. In total, 56.3% SNVs were transversions and 43.7% SNVs were transitions. Of the transitions, 48 and 52% were A/G and T/C transitions, respectively. While A/C, A/T, C/G, and G/T transversions constituted 34.09, 16.31, 8.26, and, 41.34% of the cases, respectively. Also 25 triallelic positions were observed in these *Brassica* species.

**FIGURE 2:**
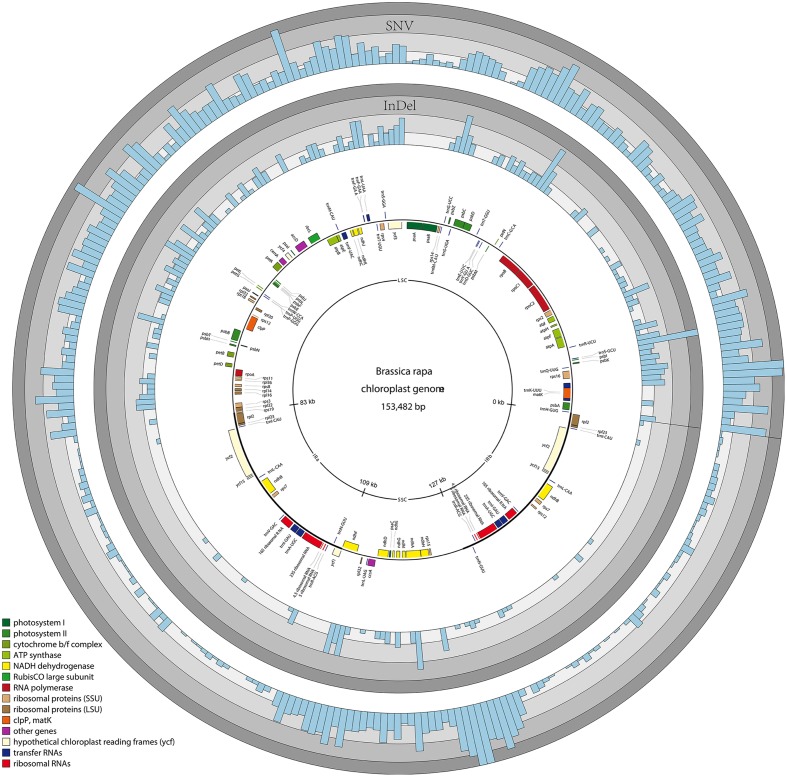
**Variability of the six economically important *Brassica species* represented over the circular map of the *B. rapa* chloroplast genome.** The two inverted repeat regions (IRa and IRb) separate the large (LSC) and small (SSC) single copy regions, respectively. The colored rings represent the density of single nucleotide variants (SNVs) (0–35) and InDels (0–16).

The average SNV density in the whole chloroplast genome was 17.2 SNVs per kb, while the average SNV density for each region (LSC, SSC, and IRs) was 21.9, 33.0 and 4.5 SNVs per kb, respectively. The IR regions showed a much lower SNV density in comparison with the chloroplast average. The average SNV density for exons (total size 87356 bp), introns (total size 12574 bp) and intergenic regions (total size 53552 bp) was 12.5, 14.0 and 25.7 SNVs per kb, respectively. About 52.0% of variant positions (1376) were found in intergenic regions. Furthermore, some genes such as *matK*, *rpoC2*, *rpoB*, *ycf2.1*, *ndhF*, and *ycf1.2*, displayed a remarkably high number of SNVs in their exons (46, 71, 39, 40, 49, and 237, respectively; **Supplementary Table S4**). A 500 bp window size was used to calculate and draw the SNVs density distribution along the chloroplast genome, to identify regions with increased SNV densities that we consider hotspots of variation of chloroplast genomes (**Figure 2**). In addition, circular maps were drawn to show the hotspots of variation of chloroplast genomes in the three diploid *Brassica* species separately (**Supplementary Figure S2**). In *B. rapa*, based on 30 chloroplast genomes, the average SNV density in the chloroplast genome was 2.2 SNVs per kb, while the average SNV density for each region (LSC, SSC, and IRs) was 3.0, 3.9, and 0.5 SNVs per kb, respectively. In *B. oleracea*, based on 17 chloroplast genomes and the reference *B. oleracea* chloroplast genome, the average SNV density in the chloroplast genome was much lower with 0.1 SNVs per kb, while the average SNV density for each region (LSC, SSC, and IRs) was 0.2, 0.2, and 0 SNVs per kb, respectively. In *B. nigra*, based on two chloroplast genomes only the average SNV density was 0.5 SNVs per kb, while the average SNV density for each region (LSC, SSC, and IRs) was 0.6, 1.3, and 0.1 SNVs per kb, respectively. In total, 343 SNVs and 125 InDels were identified in the chloroplast genomes of the 30 *B. rapa* accessions. Interestingly, the chloroplast genomes of four *B. rapa* broccoletto accessions contributed many more SNVs and InDels than chloroplast genomes of other *B. rapa* accessions (**Supplementary Table S1**).

The average InDel density in the chloroplast genome, when chloroplast genomes of all six *Brassica* species were included was 3.0 InDels per kb, while the average InDel density for each region (LSC, SSC, and IRs) was 4.6, 3.3, and 0.4 SNVs per kb, respectively. The average InDel density for exons (total size 87356 bp), introns (total size 12574 bp) and intergenic regions (total size 53552 bp) was 0.1, 4.9, and 7.3 InDel per kb, respectively. In *B. rapa*, the average InDel density in the chloroplast genome was 0.8 InDel per kb, while the average InDel density for each region (LSC, SSC, and IRs) was 1.2, 1.1, and 0.1 InDel per kb, respectively. In *B. oleracea*, the average InDel density in the chloroplast genome was 0.04 InDel per kb, while the average InDel density for each region (LSC, SSC, and IRs) was 0.06, 0.06, and 0 InDel per kb, respectively. In *B. nigra*, the average InDel density in the chloroplast genome was 0.16 InDel per kb, while the average InDel density for each region (LSC, SSC, and IRs) was 0.28, 0.06, and 0.02 InDel per kb, respectively. Analysis of the distribution of the 464 InDels revealed that 392 (84.5%) were situated in intergenic regions whereas only 72 (15.5%) were located in genes (**Supplementary Table S4**; **Supplementary Figure S3**). Sixty one out of the 72 InDels were in introns whereas the remaining 11 InDels affected the coding regions of *trnK-UUU*, *trnQ-UUG*, *rpoC2*, *trnS-UGA*, and *accD* genes. A total of 464 manually curated high-quality InDels, ranging from 1 to 38 bp (including 41 triallelic positions), were detected. Only 6 out of 464 InDels were longer than 10 bp. Furthermore, 237 InDels were single-base InDels.

### Tree Structure Using Different Data Subsets

As the single copy regions (LSC and SSC) and IRs regions have very different molecular evolutionary rates (Palmer, 1985), three subsets of the data were produced, containing either the combination of LSC and SSC, only the IR or the whole chloroplast genome sequence.

**FIGURE 3:**
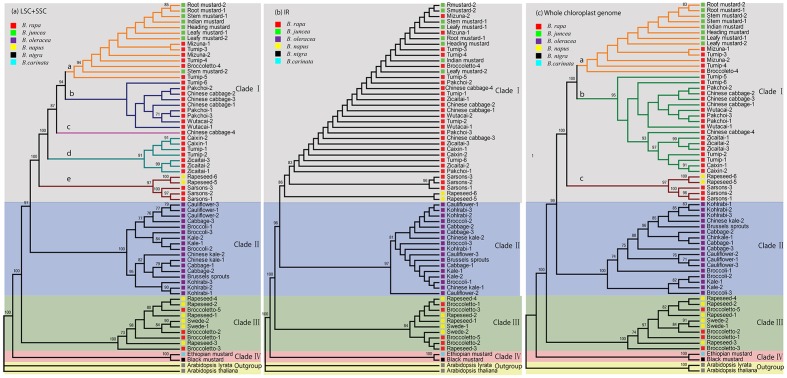
**Tree structure of *Brassica* species inferred from maximum likelihood (ML) analyses of different chloroplast genome sequences.** ML topology shown with bootstrap support values listed at each node, derived from the LSC+SSC dataset under GTR+G model **(a)**, IR dataset under GTR+I model **(b)** and whole chloroplast genome under GTR+I+G model **(c)**. Colored background of this figure indicates the four *Brassica* clades. Colored branches show subclades of clade I.

The phylogenetic relationships derived from the LSC+SSC dataset under GTR+G model, IR dataset under GTR+I model and whole chloroplast genome under GTR+I+G model are shown in **Figure 3**. The topologies estimated from ML analyses of different data subsets were similar to each other, with four clades clearly distinguished. The only differences were the poorly supported branches within lowly divergent clades (**Figure 3**). Overall, phylogenetic analyses of the three different datasets did not generate strongly supported topological conflict. The phylogenetic resolution of the LSC+SSC subset (**Figure 3a**) and the whole chloroplast genome sequences subset (**Figure 3c**) was higher than that of the IR subset (**Figure 3b**). Clade I contained the chloroplast genomes of mustards (*B. juncea*), of most *B. rapa* accessions and of two *B. napus* accessions. Clade I based on the LSC+SSC subset was divided into five subclades (subclade a–e), while divided in three subclades (subclade a–c) when based on the whole chloroplast genome. These subclade groupings do not fully agree with each other. The subclade ‘a’ in the LSC+SSC analysis, containing chloroplast genomes of all mustard accessions, and of several *B. rapa* accessions (Japanese leafy types and turnips, one Italian broccoletto, and a European turnip), generally corresponds to the subclade ‘a’ of the whole chloroplast analysis, except for the chloroplast genome of one European turnip (5). The subclade b of the whole chloroplast analysis, containing chloroplast genomes of several *B. rapa* morphotypes, corresponds to subclade b, c, and d of the LSC+SSC based analysis. Subclade ‘e’ with chloroplast genomes of sarsons (*B. rapa*) and *B. napus* is identical for the two analyses. The fact that the results of different datasets were almost identical, in addition to their corresponding internal bootstrap support, shows the reliability of the phylogenies. Moreover, ML analyses of the LSC+SSC chloroplast sequences demonstrated increased resolution in the tree topology with higher support values (**Figure 3a**) compared to the other two datasets. Thus, we mainly focus on results from the LSC+SSC region of chloroplast genome analyses.

### Phylogenetic Analysis of the *Brassica* Genus

**FIGURE 4:**
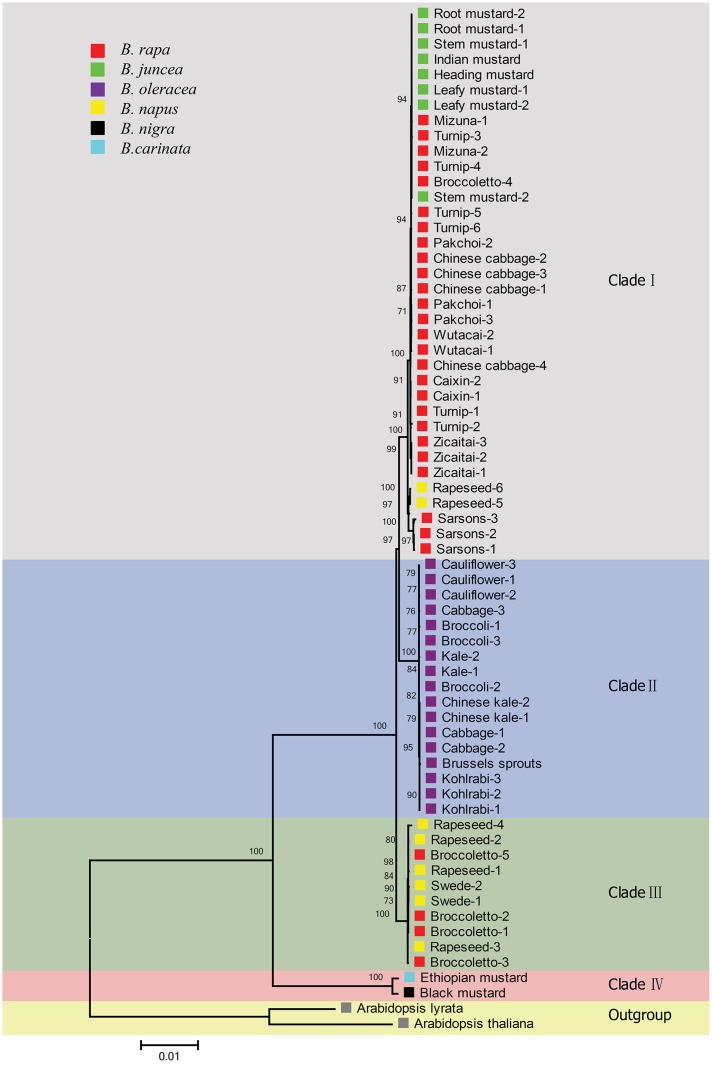
**Phylogenetic tree constructed from ML analysis using the settings of the optimal substitution model (GTR+G nucleotide model) for the alignment of single copy regions of chloroplast genomes.** Colors in the square box and the right side represent the different *Brassica* species and the separated chloroplast clades, respectively. Bootstrap support values for the clades are displayed in the corresponding branching points of the tree.

We generated a ML phylogeny from the 67 single copy LSC+SSC regions of chloroplast genomes including 60 *de novo* assembled sequences and seven previously published chloroplast genomes. The phylogenetic tree was rooted with *A. thaliana* and *A. lyrata* as outgroup and showed a clustering topology in general well supported with high bootstrap values (**Figure 4**). Our phylogenetic analyses of chloroplasts sequences indicated a clear division of the genus *Brassica* into two separate evolutionary linages, including four different clades (**Figure 4**), which was quite similar to the phylogeny using whole chloroplast sequences (**Supplementary Figure S4**).

One of these two separate linages contained three clades formed by the chloroplast genomes of *B. rapa*, *B. oleracea*, *B. napus* and *B. juncea* accessions (**Figure 4**). The clade I (strongly supported with 100% BP), contained the chloroplast genomes of 26 *B. rapa* accessions representing all different *B. rapa* morphotypes, together with chloroplast genomes of two *B. napus* accessions and of all the eight *B. juncea* accessions. Furthermore, the chloroplast genomes of these two *B. napus* accessions were grouped with those of three *B. rapa* (sarsons) in this clade. The chloroplast genomes of all the *B. juncea* accessions were grouped with those of the *B. rapa* accessions in clade I, supporting that *B. rapa* is the maternal ancestor of *B. juncea.* In clade II, the chloroplast genomes of all the 17 *B. oleracea* accessions we examined were placed together. Interestingly, clade III in the phylogenetic tree is composed of the chloroplast genomes of six *B. napus* and four *B. rapa* broccoletto accessions, significantly supported by bootstrap values. The chloroplast genomes of the six *B. napus* accessions that include both rapeseed and swede accessions, were closely related to those of the four broccoletto accessions and less related to those of the other two rapeseed *B. napus* accessions that were placed in clade I, close to the chloroplast genomes of the *B. rapa* sarsons. Clade IV represents the other lineage, and corresponds to *B. nigra* (black mustard) and *B. carinata* (Ethiopian mustard) chloroplast genomes, supporting that *B. nigra* is the maternal ancestor of *B. carinata*. The branch length of the tree indicated that *B. nigra* and *B. carinata* chloroplast genomes are more distant from *B. oleracea*, *B. napus*, *B. juncea* and *B. rapa* chloroplast genomes than the chloroplast genomes of these four species are from each other, suggesting much earlier divergence of *B. nigra* from the other diploid Brassica’s.

### Estimation of Divergence Times in the *Brassica* Genus

**FIGURE 5:**
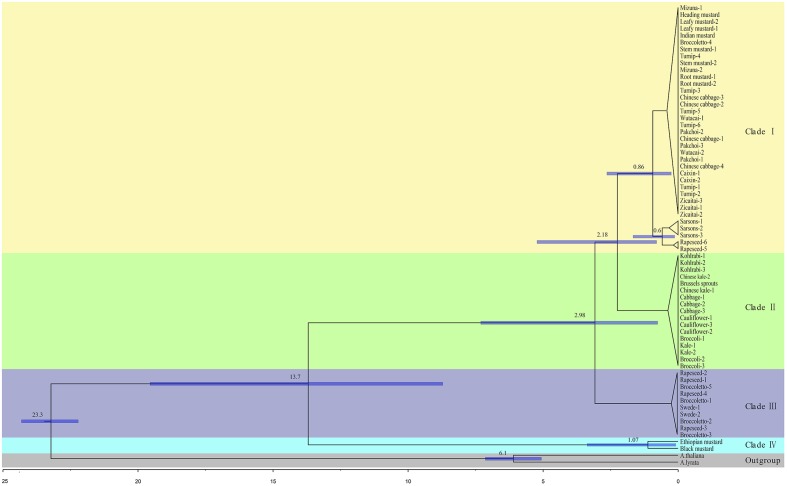
***Brassica* chronogram inferred using BEAST program from sequence alignment of chloroplast genomes using *Arabidopsis* as outgroup.** The root age was constrained by a normal distribution with a mean of 23.35 Mya. In addition, the divergence age of *A. thaliana* and *A. lyrata* was set at 5.97 Mya. Dating for the clusters, which branching pattern could not be resolved in the ML analysis (**Figure 4**), was considered to be unreliable and is not shown here. Divergence times are indicated with 95% confidence intervals.

To estimate when separation of these chloroplast lineages in *Brassica* occurred, we used two calibration points of speciation events calibrated by valid fossil evidence (Hohmann et al., 2015). The results of our dating experiments are presented in **Figure 5**.

Our results indicate that, the ancestors of the current major *Brassica* groups were generated in several speciation events that started with a first radiation approximately 13.7 Mya separating clade IV (*B. nigra* and *B. carinata*) from the rest of *Brassica* species. *B. nigra* and *B. carinata* species diverged from each other 1.07 Mya. In a second period of speciation, the clade III (broccoletto and *B. napus*) separated from clade I (*B. rapa*, *B. juncea* and *B. napus*) and clade II (*B. oleracea*) at 2.98 Mya. In a third period, approximately 2.18 Mya, the chloroplast line shared by clade I (*B. rapa*, *B. juncea* and *B. napus*) diverged from clade II (*B. oleracea*). Later on, the Sarson chloroplast lines diverged from the *B. rapa* cluster approximately 0.86 Mya.

## Discussion

As plant cells contain multiple copies of chloroplast genomes (Pyke, 1999), low coverage total genome sequencing data are useful for both nuclear marker acquisition as well as for the assembly of chloroplast genomes (Tangphatsornruang et al., 2010; Nock et al., 2011). Here we demonstrate that low coverage resequencing data (at 1x nuclear genome coverage) can be used for a complete *de novo* assembly of the chloroplast genomes of *Brassica* species. Despite the fact that complete chloroplast genome sequences can provide a wealth of information to study the origin of photosynthetic eukaryotes (Chu et al., 2004), few *Brassica* chloroplast genomes have been published during the past decade (Wu et al., 2012; Hao et al., 2014; Prabhudas et al., 2015). In this study, the assembly of 60 complete chloroplast genomes representing the three diploid and three amphidiploid *Brassica* species (**Supplementary Table S1**) has certainly added new and valuable information to solve important controversies on the evolution of the genus (Warwick and Black, 1991). The assembled genomes allowed us to derive a highly reliable phylogenetic tree, rooted with *Arabidopsis* as outgroup (**Figure 4**).

Due to the uniparental inheritance and absence of recombination, the chloroplast genome is phylogenetically linear over generations, changing only by occasional mutations. In this research, a total of 2646 SNVs and 464 InDels were identified, which rendered patterns of distribution across species compatible with the topology of the phylogenetic tree (**Figure 3**), providing an independent support to our proposal. In the chloroplast genomes of all the *B. rapa* accessions we detected, 343 SNVs and 125 InDels, whereas only 16 SNVs and 6 InDels were detected within the chloroplast genomes of 17 *B. oleracea* accessions. This observation agrees with the results from previous nuclear RFLP and chloroplast SSR analysis that indicated more genetic diversity in chloroplast genomes of *B. rapa*’s than of *B. oleracea’s* (Song et al., 1990; Allender and King, 2010). Several coding regions accumulated a higher number of variants compared with the average regions of the genome. Furthermore, several specific genes accumulated simultaneously a high number of SNVs and at least one InDel in their coding region (**Supplementary Table S4**), suggesting that these genes may function as general hotspots of natural genetic variation in *Brassica* chloroplast genomes. Meanwhile, the specific variations can be used as markers for haplotype identification to distinguish different cytoplasms. Our preliminary data indicate that the variations in IRs are much lower than in LSC and SSC regions. This difference in evolutionary rate has previously been attributed to conservation of the ribosomal RNA genes, which occupy about one-third of the IR region of the chloroplast genomes (Palmer, 1985).

As one of our aims is to see whether the amphidiploid species are the result of a single hybridization event or from multiple events, we first tried to distinguish the chloroplast genomes both within and between diploid *Brassica* species. We investigated the chloroplast genomes of 30 *B. rapa* accessions and 17 *B. oleracea* accessions, and found that the chloroplast genomes of *B. rapa* are separated into two clades as four broccoletto’s have clearly different chloroplast genomes compared to those of all the other *B. rapa* accessions. This means that we revealed two different chloroplast genome types in *B. rapa*, while we only identified one chloroplast genome type in *B. oleracea*. Interestingly, these four broccoletto accessions group with the other *B. rapa* accessions in nuclear phylogenies (including the broccoletto with the Clade I chloroplast genome) (Zhao et al., 2005) and so it is puzzling how broccoletto obtained another chloroplast genome. The broccoletto morphotype originates from Italy, where also many wild *Brassica* species can be found (Hammer et al., 1992), including many wild C9 species, but also *B. repanda* with *n* = 10. It is likely that broccoletto has been cultivated alongside wild *Brassica* species indigenous to Italy, providing the necessary opportunities for inter-specific crosses to occur (Allender and King, 2010). After interspecific crosses with wild Brassica’s as female parent, natural backcrosses may have reverted the nuclear genome to *B. rapa* broccoletto. The extensive genome synteny between *Brassica* species (Chalhoub et al., 2014; Liu et al., 2014), may have facilitated the generation of percentages of viable offspring from interspecific crosses, through chromosome pairing of syntenic regions in meiosis. This is in agreement with the hypotheses raised by Allender and King (2010). Another explanation is that, after interspecific crosses between wild Brassica’s and *B. rapa*, amphidiploids were generated due to spontaneous chromosome doubling of the hybrids (Zhou et al., 2016). Also these amphidiploids may have reverted to the diploid *B. rapa* genome trough many generations of backcrosses and resulting loss of the cytoplasm donor genome chromosomes (Gupta et al., 2015).

As chloroplasts in *Brassica* are exclusively maternally inherited (Scott and Wilkinson, 1999), chloroplast phylogenies represent the maternal lineage of the amphidiploid hybrids only, and each amphidiploid species will cluster with its maternal ancestor. The chloroplast genomes of all eight *B. juncea* accessions were clustering with the chloroplast genomes of *B. rapa* accessions in subclade ‘I-a’ (Japanese leafy types and turnips, plus one broccoletto), in agreement with the finding that *B. rapa* is the ancestral maternal parent of amphidiploid *B. juncea* (Palmer et al., 1983). Chloroplast genomes of *B. carinata* and *B. nigra* were grouped in clade IV, indicating that *B. carinata* has the cytoplasm origin of *B. nigra*. The fact that *B. napus* has two chloroplast types (I-e and III), suggests that the studied accessions may have been derived from independent hybridization events involving different female parental accessions, one with a broccoletto and the other event with a sarson as parent. It is not unexpected that *B. napus* is derived from a cross with a sarson like morphotype, as both are selected for their seeds and oil content. It is likely that both the sarsons and broccoletto would have been cultivated alongside *B. oleracea* crops such as kales, cabbages, and broccolis (Schery, 1972), providing the necessary opportunities for inter-specific crosses to occur. It is also possible that some *B. oleracea* hybridized with wild *B. rapa* as female, which was not sampled in our study, and then transferred cytoplasm to broccoletto through introgression. However, we did not find any amphidiploid hybrids grouped with *B. oleracea*, demonstrating that *B. oleracea* is not the donor to any amphidiploid species. This fits with the observation that in interspecific crosses, *B. oleracea* can only be used as male parent.

Estimations of the age of the *Brassica* genus in literature are scarce and controversial (Lysak et al., 2005; Cheung et al., 2009; Beilstein et al., 2010; Hohmann et al., 2015). Caution must be taken to choose correct anchoring points in order to produce correct time lines for the species we studied. In this study, we used the time-calibrated divergence time of 23.35 Mya for the radiation of *Arabidopsis* and *Brassica*, estimated from chloroplast sequence data involving four fossil constraints (Hohmann et al., 2015) and not of 43 Mya, as was the estimation based on fossil evidence of a *Thlaspi primaevum* (Brassicaceae, dated at 20.8–29.2 Mya) specimen. The main argumentation to reject the estimation based on the *Thlaspi* fossil (Wing, 1987; Franzke et al., 2010) is that identification of this genus has been difficult when relying only on morphological characteristics (Mummenhoff et al., 1997; Hohmann et al., 2015).

The calibrated tree presented in **Figure 5** clearly depicts an evolutionary scenario with three main periods of speciation, which can be considered a first attempt to date the evolutionary history of the members of the *Brassica* genus using chloroplast genomes. Previous studies of diversification dates based on nuclear and chloroplast markers estimate that *B. rapa* and *B. oleracea* diverged from *B. nigra* 7.9 Mya (Lysak et al., 2005) or 20 Mya (Arias et al., 2014), respectively, while our data shows the divergence at 13.7 Mya. Furthermore, the speciation of clade III from clade I and clade II 2.98 Mya had never been estimated based on nuclear genomes. At approximately 2.18 Mya, the chloroplast line shared by clade I (*B. rapa*, *B. juncea* and *B. napus*) diverged from clade II (*B. oleracea*), which is intermediate compared to previous studies estimating that the genomes of *B. rapa* and *B. oleracea* diverged from each other 3.7 Mya (Inaba and Nishio, 2002) or 1.37–0.12 Mya (Cheung et al., 2009).

In summary, we conclude that the chloroplast genomes of the six economically important Brassica species are organized in at least four different clades. *B. rapa* chloroplast genomes display most variation and are represented by both clade I with five subclades and clade III. *B. juncea* has the chloroplast origin of clade I-a *B. rapa’s*, while *B. carinata* has the chloroplast origin of clade IV *B. nigra. B. napus* has two chloroplast origins, one corresponding to clade I-e (the *B. rapa* sarsons) and the other one to clade III (a *B. rapa* broccoletto accession). In addition, we present estimates of divergence time for the different chloroplast genomes. Knowledge of the parental genotypes of the amphidiploid *Brassica* species is very relevant to design further crop improvement strategies of especially *B. napus* via interspecific crosses.

## Author Contributions

PL extracted DNA, analyzed the data, and drafted the manuscript. FL prepared samples. SjZ, SfZ, HZ, and XW provided advices on our analysis. RS, TB, and GB designed and supervised the work, and contributed to writing the manuscript.

## Conflict of Interest Statement

The authors declare that the research was conducted in the absence of any commercial or financial relationships that could be construed as a potential conflict of interest.

## Footnotes

1 http://brassicadb.org/brad/datasets/pub/CPassem/

## Supplementary Material

**FIGURE S1 | K-mer frequency histograms of *Brassica* species.** Chloroplast genomes of six accessions were shown as examples. The two expected peaks for k-mers derived from the chloroplast genome sequences are clearly visible as sharp peaks and noted in this figure.

**FIGURE S2 | Variability of chloroplast genomes of three diploid *Brassica* species represented over the circular map.** The two inverted repeat regions (IRa and IRb) separate the large (LSC) and small (SSC) single copy regions, respectively. The colored rings represent the density of SNVs (0–16) and InDels (0–8).

**FIGURE S3 | Sequence comparison of the eight chloroplast genomes generated with mVISTA.** Gray arrows indicate the position and direction of each gene. Areas colored in blue and red indicate genic and non-coding regions, respectively. Black lines designate regions of sequence identity with *B. rapa*, using a 50% identity cutoff. Dashed rectangles indicate highly divergent regions of these species compared to *B. rapa*.

**FIGURE S4 | Phylogenetic tree constructed from ML analysis using the whole chloroplast genomes.**

**TABLE S1 | Data summary of the chloroplast genomes of several *Brassica* species.**

**TABLE S2 | SNVs identified in chloroplast genomes of sequenced accessions.**

**TABLE S3 | InDels identified in chloroplast genomes of sequenced accessions.**

**TABLE S4 | Genes in the *Brassica* chloroplast genome containing SNVs and InDels.**
