# Supplementary figures and images for: A Phylogenetic Analysis of Chloroplast Genomes Elucidates the Relationships of the Six Economically Important Brassica Species Comprising the Triangle of U
Source: Front Plant Sci. 2017 Feb 2;8:111. doi: 10.3389/fpls.2017.00111 (PMC5288352; doi:10.3389/fpls.2017.00111)

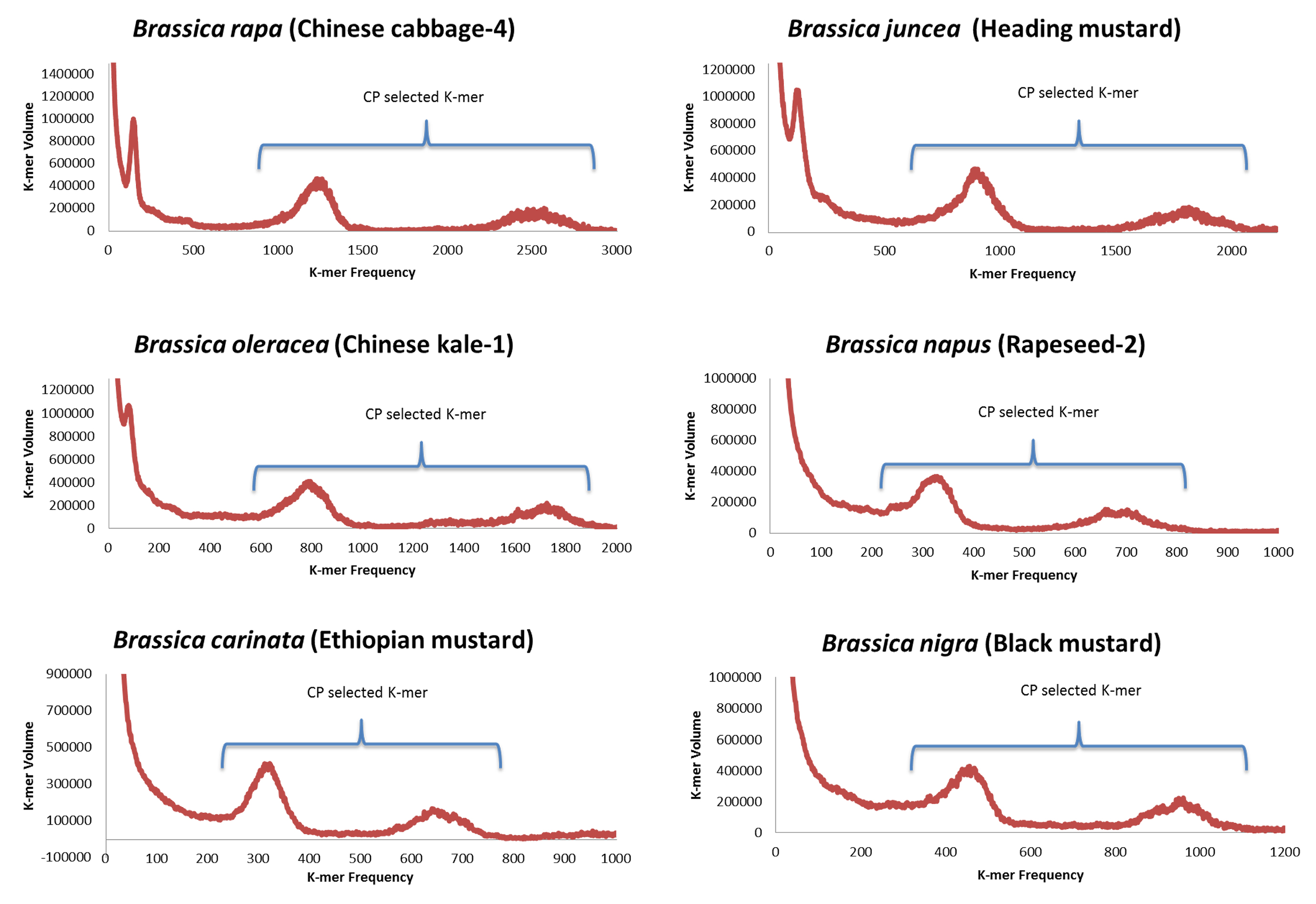

Supplement: Supplementary file 1 [file Image_1.JPEG]

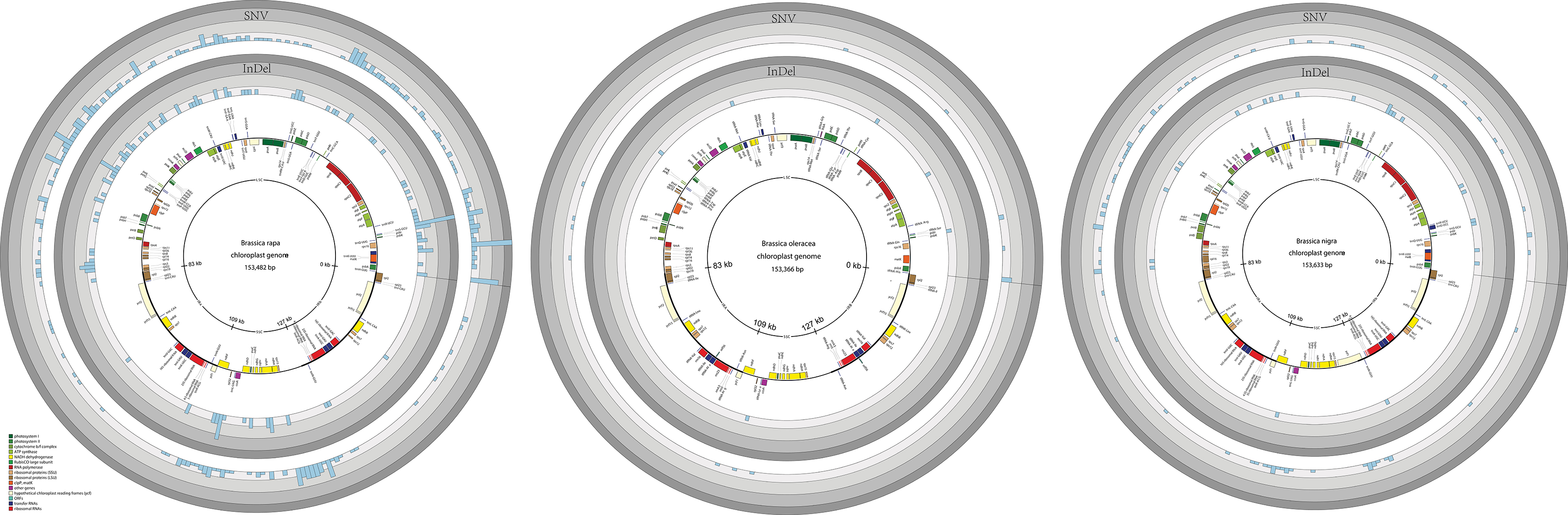

Supplement: Supplementary file 2 [file Image_2.JPEG]

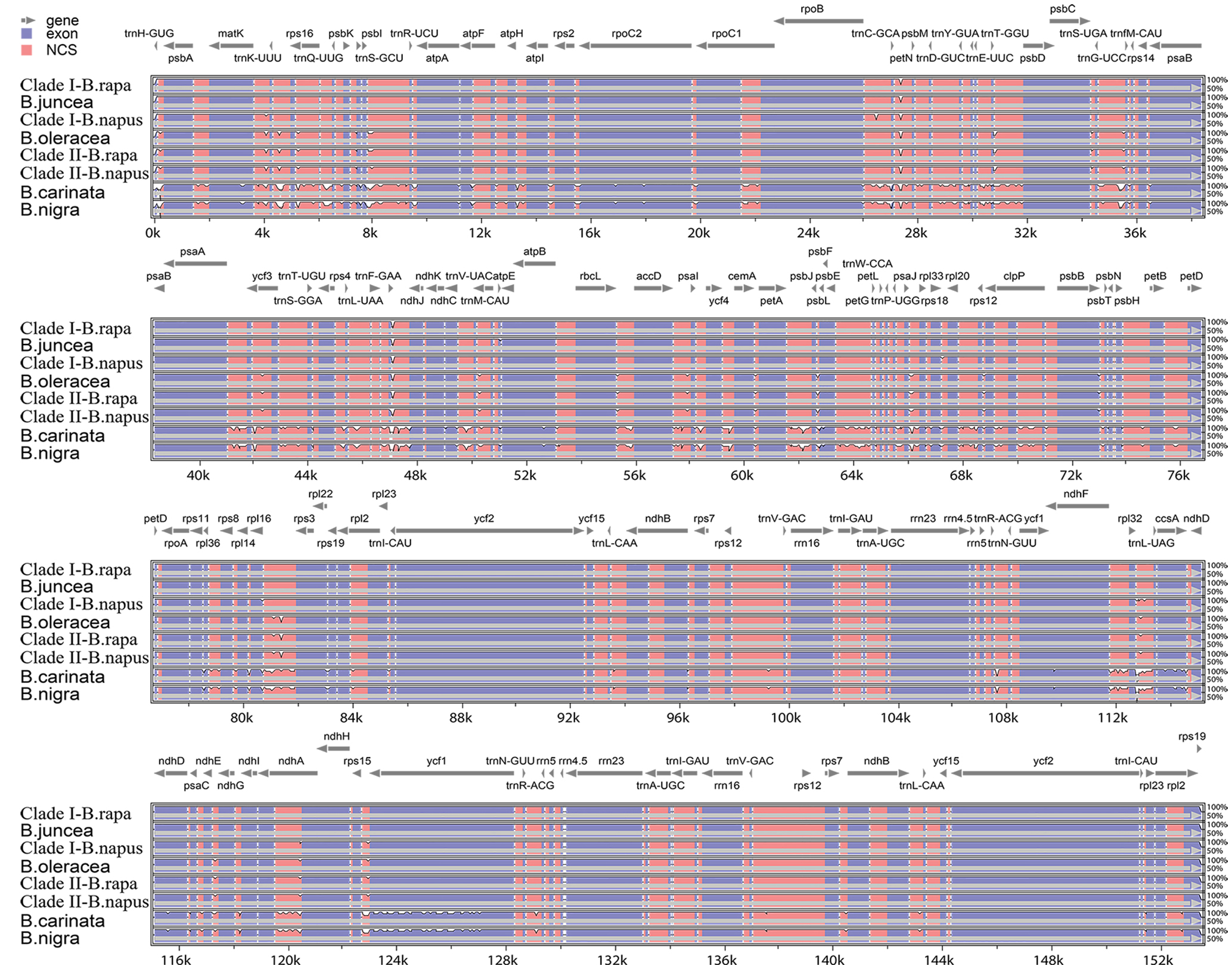

Supplement: Supplementary file 3 [file Image_3.JPEG]

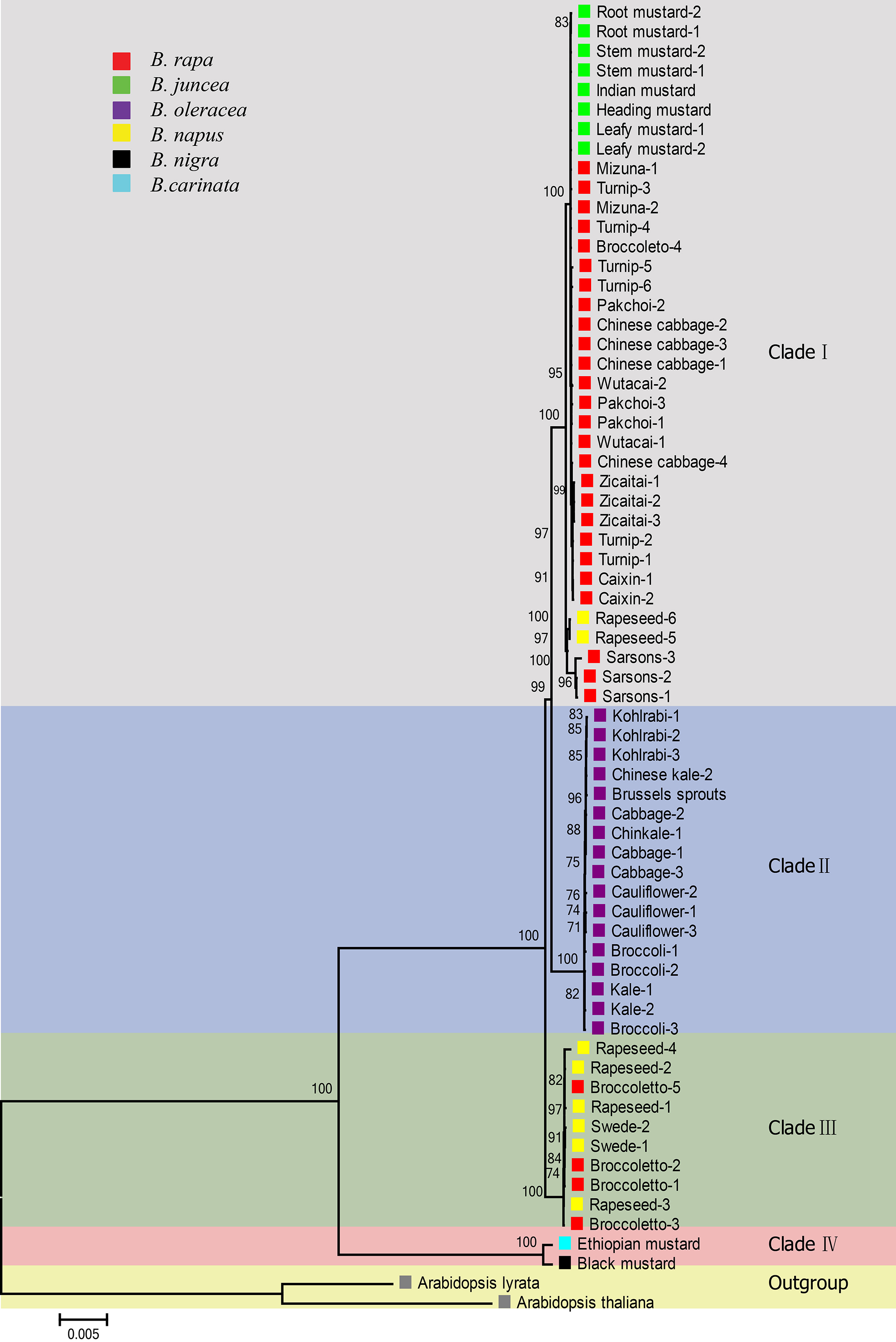

Supplement: Supplementary file 4 [file Image_4.JPEG]
