# Supplementary material for: A Phylogenetic Analysis of Chloroplast Genomes Elucidates the Relationships of the Six Economically Important Brassica Species Comprising the Triangle of U
Source: Front Plant Sci. 2017 Feb 2;8:111. doi: 10.3389/fpls.2017.00111 (PMC5288352; doi:10.3389/fpls.2017.00111)
Supplement: Supplementary file 5 [file Table_1.DOCX]

**Supplementary** **Table 1** Data summary of the chloroplast genome of several *Brassica* species

| Species | Common Name | Accession | Origion | SNPs | InDels | Mapped Reads | Mean Coverage | Genome size |
| --- | --- | --- | --- | --- | --- | --- | --- | --- |
| *Brassica rapa* | Chinese cabbage-1 | A237 | China | 6 | 0 | 599734 | 320 | 153482 |
| *Brassica rapa* | Chinese cabbage-2 | A548 | China | 6 | 0 | 432871 | 230 | 153482 |
| *Brassica rapa* | Chinese cabbage-3 | A266 | China | 6 | 1 | 436085 | 240 | 153478 |
| *Brassica rapa* | Chinese cabbage-4 | ER1739 | Korea | 0^a^ | 0 | 348552 | 340 | 153482 |
| *Brassica rapa* | Pak-choi-1 | ER1675 | China | 7 | 1 | 951745 | 930 | 153486 |
| *Brassica rapa* | Pak-choi-2 | ER1687 | China | 6 | 0 | 613133 | 600 | 153482 |
| *Brassica rapa* | Pak-choi-3 | ER1696 | China | 7 | 1 | 491042 | 480 | 153485 |
| *Brassica rapa* | Turnip-1 | ER1747 | China | 7 | 1 | 560837 | 550 | 153481 |
| *Brassica rapa* | Turnip-2 | ER1744 | China | 7 | 1 | 250264 | 240 | 153484 |
| *Brassica rapa* | Turnip-3 | DF10C088 (CGN15201) | Japan | 11 | 5 | 1272784 | 610 | 153488 |
| *Brassica rapa* | Turnip-4 | DF10C089 (CGN06721) | Japan | 9 | 7 | 2752712 | 1300 | 153482 |
| *Brassica rapa* | Turnip-5 | FS147 (CGN06710) | Europe | 7 | 2 | 401052 | 200 | 153477 |
| *Brassica rapa* | Turnip-6 | DF10C090 (CGN06688) | Europe | 5 | 0 | 3659487 | 1700 | 153482 |
| *Brassica rapa* | Wutacai-1 | P223 | China | 7 | 2 | 497365 | 270 | 153480 |
| *Brassica rapa* | Wutacai-2 | P138 | China | 7 | 1 | 427965 | 230 | 153485 |
| *Brassica rapa* | Caixin-1 | P045 | China | 8 | 0 | 579861 | 310 | 153481 |
| *Brassica rapa* | Caixin-2 | P048 | China | 8 | 1 | 266766 | 140 | 153467 |
| *Brassica rapa* | Zicaitai-1 | DF10C070 | China | 12 | 1 | 1809699 | 850 | 153483 |
| *Brassica rapa* | Zicaitai-2 | DF10C071 | China | 13 | 1 | 4441270 | 2100 | 153483 |
| *Brassica rapa* | Zicaitai-3 | P058 | China | 13 | 1 | 752767 | 410 | 153483 |
| *Brassica rapa* | Mizuna-1 | P220 | Japan | 10 | 4 | 528151 | 290 | 153481 |
| *Brassica rapa* | Mizuna-2 | A525 | Japan | 10 | 5 | 647352 | 350 | 153496 |
| *Brassica rapa* | Broccoletto-1 | 14CELS075 | Italy | 247 | 104 | 353999 | 230 | 153507 |
| *Brassica rapa* | Broccoletto-2 | 14CELS076 | Italy | 249 | 101 | 433100 | 280 | 153507 |
| *Brassica rapa* | Broccoletto-3 | 14CELS077 | Italy | 242 | 102 | 610271 | 400 | 153507 |
| *Brassica rapa* | Broccoletto-4 | FS002 (CGN06824) | Italy | 8 | 6 | 645595 | 330 | 153485 |
| *Brassica rapa* | Broccoletto-5 | FS003 (CGN06825) | Italy | 250 | 98 | 953017 | 490 | 153489 |
| *Brassica rapa* | Sarsons-1 | DF10C092 | India | 84 | 20 | 2373787 | 1200 | 152982 |
| *Brassica rapa* | Sarsons-2 | A545 | India | 87 | 24 | 414466 | 220 | 153037 |
| *Brassica rapa* | Sarsons-3 | DF10C081 | India | 87 | 27 | 6806867 | 3200 | 153036 |
| *Brassica juncea* | Heading mustard | ER1708 | China | 9 | 3 | 788794 | 770 | 153483 |
| *Brassica juncea* | Root mustard-1 | ER1709 | China | 10 | 4 | 1069144 | 1000 | 153802 |
| *Brassica juncea* | Root mustard-2 | ER1733 | China | 10 | 4 | 625668 | 610 | 153489 |
| *Brassica juncea* | Stem mustard-1 | ER1710 | China | 10 | 4 | 785426 | 770 | 153483 |
| *Brassica juncea* | Stem mustard-2 | ER1712 | China | 9 | 4 | 1010910 | 990 | 153482 |
| *Brassica juncea* | Leafy mustard-1 | ER1713 | China | 9 | 3 | 617628 | 600 | 153483 |
| *Brassica juncea* | Leafy mustard-2 | ER1711 | China | 10 | 7 | 1782434 | 1700 | 153485 |
| *Brassica juncea* | Indian mustard | KT581449^b^ | India | - | - | - | - | 153483 |
| *Brassica napus* | Swede-1 | ER1715 | China | 252 | 101 | 563232 | 550 | 153428 |
| *Brassica napus* | Swede-2 | ER1748 | China | 250 | 92 | 209114 | 210 | 153454 |
| *Brassica napus* | Rapeseed-1 | ER1719 | Canada | 252 | 96 | 463210 | 460 | 153479 |
| *Brassica napus* | Rapeseed-2 | ER1720 | Canada | 252 | 98 | 335203 | 330 | 153453 |
| *Brassica napus* | Rapeseed-3 | KP161617.1^b^ | China | - | - | - | - | 153454 |
| *Brassica napus* | Rapeseed-4 | GQ861354.1^b^ | China | - | - | - | - | 152860 |
| *Brassica napus* | Rapeseed-5 | KM454973.1^b^ | China | - | - | - | - | 153531 |
| *Brassica napus* | Rapeseed-6 | KJ872515.1^b^ | China | - | - | - | - | 153533 |
| *Brassica oleracea* | Broccoli-1 | ER1721 | China | 293 | 95 | 480995 | 470 | 153364 |
| *Brassica oleracea* | Broccoli-2 | WBol003 | Europe | 293 | 94 | 6074037 | 4000 | 153363 |
| *Brassica oleracea* | Broccoli-3 | WBol014 | Europe | 288 | 93 | 6326883 | 4100 | 153581 |
| *Brassica oleracea* | Kale-1 (Purple) | ER1722 | China | 294 | 89 | 261511 | 260 | 153363 |
| *Brassica oleracea* | Kale-2 | ER1723 | China | 297 | 90 | 423713 | 420 | 153362 |
| *Brassica oleracea* | Chinese kale-1 | ER1742 | China | 296 | 90 | 821201 | 810 | 153365 |
| *Brassica oleracea* | Chinese kale-2 | ER1714 | China | 298 | 92 | 807127 | 790 | 153417 |
| *Brassica oleracea* | Cauliflower-1 | WBol052 | Europe | 290 | 94 | 6922360 | 4500 | 153366 |
| *Brassica oleracea* | Cauliflower-2 | WBol055 | Europe | 292 | 96 | 5719663 | 3700 | 153367 |
| *Brassica oleracea* | Cauliflower-3 | WBol053 | Europe | 287 | 94 | 6293448 | 4100 | 153581 |
| *Brassica oleracea* | Kohlrabi-1 | WBol039 | Europe | 292 | 91 | 5316311 | 3500 | 153364 |
| *Brassica oleracea* | Kohlrabi-2 | WBol104 | Europe | 294 | 91 | 5625291 | 3700 | 153364 |
| *Brassica oleracea* | Kohlrabi-3 | WBol106 | Europe | 294 | 87 | 5823426 | 3800 | 153265 |
| *Brassica oleracea* | Brussels sprouts | ER1740 | China | 296 | 87 | 364620 | 360 | 153365 |
| *Brassica oleracea* | Cabbage-1 | ER1725 | China | 295 | 90 | 660292 | 650 | 153364 |
| *Brassica oleracea* | Cabbage-2 | WBol111 | Europe | 289 | 92 | 5217324 | 3400 | 153365 |
| *Brassica oleracea* | Cabbage-3 (purple) | ER1724 | China | 297 | 91 | 392230 | 390 | 153365 |
| *Brassica carinata* | Ethiopian mustard | ER1728 | Ethiopia | 2139 | 307 | 212680 | 220 | 153694 |
| *Brassica nigra* | Black mustard | ER1755 | Ethiopia | 2193 | 309 | 304570 | 310 | 153700 |
| *Arabidopsis thaliana* | - | NC_000932.1^b^ | - | - | - | - | - | 154478 |
| *Arabidopsis lyrata* | - | LN877383.1^b^ | - | - | - | - | - | 154905 |

NOTE.— The reference genome sequence of the *Brassica rapa* subsp. *pekinensis* (Chiffu) chloroplast was used to detect SNVs and InDels.

a There was a nonconcordant position (116457) between the Sanger reference sequence of the *Brassica rapa* subsp. *pekinensis* chloroplast (G) and the chloroplast genome resequenced here (A). This discrepancy that may be attributed to Sanger sequencing error in the reference genome were not used in further comparative analyses.

b Those genomes were obtained from original published paper and used in our analysis.
