# Supplementary material for: A Phylogenetic Analysis of Chloroplast Genomes Elucidates the Relationships of the Six Economically Important Brassica Species Comprising the Triangle of U
Source: Front Plant Sci. 2017 Feb 2;8:111. doi: 10.3389/fpls.2017.00111 (PMC5288352; doi:10.3389/fpls.2017.00111)
Supplement: Supplementary file 8 [file Table_4.DOCX]

**Supplementary Table 4** Genes in the chloroplast *Brassica* genome containing SNVs and InDels

| **Gene Name** | **Protein Name** | **Number of SNVs** | | **Number of InDels** | | **Intron size** | **Exon size** |
| --- | --- | --- | --- | --- | --- | --- | --- |
|  |  | **Intron** | **Exon** | **Intron** | **Exon** |  |  |
| *psbA* | photosystem II protein D1 | - | 12 | - | - | 0 | 1059 |
| *matK* | maturase K | - | 46 | - | - | 0 | 1572 |
| *trnK-UUU* | tRNA-Lys | - | - | - | 1 |  |  |
| *rps16* | ribosomal protein S16 | 22 | 4 | 11 | - | 866 | 238 |
| *trnQ-UUG* | tRNA-Gln | - | - | - | 1 |  |  |
| *psbK* | photosystem II protein K | - | 2 | - | - | 0 | 183 |
| *psbI* | photosystem II protein I | - | 1 | - | - | 0 | 108 |
| *atpA* | ATP synthase CF1 alpha subunit | - | 24 | - | - | 0 | 1521 |
| *atpF* | ATP synthase CF0 subunit I | 17 | 9 | 4 | - | 715 | 552 |
| *atpH* | ATP synthase CF0 subunit III | - | 2 | - | - | 0 | 243 |
| *atpI* | ATP synthase CF0 subunit IV | - | 10 | - | - | 0 | 747 |
| *rps2* | ribosomal protein S2 | - | 6 | - | - | 0 | 708 |
| *rpoC2* | RNA polymerase beta" subunit | - | 71 | - | 5 | 0 | 4131 |
| *rpoC1* | RNA polymerase beta | 23 | 26 | 6 | - | 761 | 2064 |
| *rpoB* | RNA polymerase beta subunit | - | 39 | - | - | 0 | 3216 |
| *petN* | cytochrome b6/f complex subunit VIII | - | 1 | - | - | 0 | 87 |
| *psbD* | photosystem II protein D2 | - | 5 | - | - | 0 | 1059 |
| *psbC* | photosystem II CP43 chlorophyll apoprotein | - | 9 | - | - | 0 | 1419 |
| *trnS-UGA* | tRNA-Ser | - | - | - | 1 |  |  |
| *rps14* | ribosomal protein S14 | - | 6 | - | - | 0 | 300 |
| *psaB* | photosystem I P700 apoprotein A2 | - | 11 | - | - | 0 | 2202 |
| *psaA* | photosystem I P700 apoprotein A1 | - | 13 | - | - | 0 | 2250 |
| *ycf3* | hypothetical chloroplast RF34 | 8 | 4 | 8 | - | 784 | 378 |
| *rps4* | ribosomal protein S4 | - | 8 | - | - | 0 | 603 |
| *trnL-UAA* | tRNA-Leu | 7 | - | 1 | - | 311 | 87 |
| *trnF-GAA* | tRNA-Phe | - | 3 | - | - | 0 | 127 |
| *ndhJ* | NADH-plastoquinone oxidoreductase subunit J | - | 2 | - | - | 0 | 474 |
| *ndhK* | NADH-plastoquinone oxidoreductase subunit K | - | 10 | - | - | 0 | 675 |
| *ndhC* | NADH-plastoquinone oxidoreductase subunit 3 | - | 7 | - | - | 0 | 360 |
| *trnV-UAC* | tRNA-Val | 8 | - | 4 | - | 607 | 76 |
| *atpE* | ATP synthase CF1 epsilon subunit | - | 3 | - | - | 0 | 396 |
| *atpB* | ATP synthase CF1 beta subunit | - | 27 | - | - | 0 | 1494 |
| *rbcL* | ribulose 1,5-bisphosphate carboxylase/oxygenase large subunit | - | 21 | - | - | 0 | 1437 |
| *accD* | Acetyl-CoA carboxylase carboxyltransferase beta subunit | - | 27 | - | 3 | 0 | 1452 |
| *ycf4* | photosystem I assembly protein ycf4 | - | 10 | - | - | 0 | 552 |
| *cemA* | envelope membrane protein | - | 3 | - | - | 0 | 687 |
| *petA* | cytochrome f | - | 8 | - | - | 0 | 960 |
| *psbL* | photosystem II protein L | - | 1 | - | - | 0 | 114 |
| *psbE* | photosystem II cytochrome b559 alpha subunit | - | 3 | - | - | 0 | 249 |
| *psaJ* | photosystem II protein J | - | 1 | - | - | 0 | 132 |
| *rpl33* | ribosomal protein L33 | - | 3 | - | - | 0 | 198 |
| *rps18* | ribosomal protein S18 | - | 2 | - | - | 0 | 303 |
| *rpl20* | ribosomal protein L20 | - | 6 | - | - | 0 | 351 |
| *rps12a* | ribosomal protein S12 | - | 1 | - | - | 0 | 114 |
| *clpP* | clp protease proteolytic subunit | 40 | 8 | 12 | - | 1501 | 594 |
| *psbB* | cytochrome b6 | - | 18 | - | - | 0 | 1524 |
| *psbT* | photosystem II protein T | - | 1 | - | - | 0 | 99 |
| *psbH* | photosystem II phosphoprotein | - | 7 | - | - | 0 | 219 |
| *petB* | cytochrome b6 | - | 3 | - | - | 0 | 486 |
| *petD* | cytochrome b6/f complex subunit IV | - | 2 | - | - | 0 | 522 |
| *rpoA* | RNA polymerase alpha subunit | - | 12 | - | - | 0 | 981 |
| *rps11* | ribosomal protein S11 | - | 6 | - | - | 0 | 414 |
| *rpl36* | ribosomal protein L36 | - | 1 | - | - | 0 | 111 |
| *rps8* | ribosomal protein S8 | - | 5 | - | - | 0 | 402 |
| *rpl14* | ribosomal protein L14 | - | 11 | - | - | 0 | 366 |
| *rpl16* | ribosomal protein L16 | - | 7 | 2 | - | 0 | 435 |
| *rps3* | ribosomal protein S3 | - | 3 | - | - | 0 | 654 |
| *rpl22* | ribosomal protein L22 | - | 12 | - | - | 0 | 480 |
| *rps19.1* | ribosomal protein S19 | - | 4 | - | - | 0 | 276 |
| *rpl2.1* | ribosomal protein L2 | 3 | 0 | - | - | 684 | 825 |
| *rpl23.1* | ribosomal protein L23 | - | 1 | - | - | 0 | 279 |
| *ycf2.1* | hypothetical chloroplast RF21 | - | 40 | - | - | 0 | 6861 |
| *ycf15.1* | hypothetical chloroplast RF15 | - | 4 | - | - | 0 | 231 |
| *ndhB.1* | NADH-plastoquinone oxidoreductase subunit 2 | - | 2 | - | - | 679 | 1530 |
| *rrn16* | 16S ribosomal RNA | - | 1 | - | - | 0 | 1491 |
| *trnI-GAU* | tRNA-Ile | 1 | - | - | - | 804 | 77 |
| *trnA-UGC* | tRNA-Ala | 3 | 1 | - | - | 802 | 71 |
| *rrn23* | 23S ribosomal RNA | - | 5 | - | - | 0 | 2810 |
| *rrn4.5* | 4.5 ribosomal RNA | - | 1 | - | - | 0 | 103 |
| *ycf1.1* | hypothetical chloroplast RF19 | - | 9 | - | - | 0 | 1029 |
| *ndhF* | NADH-plastoquinone oxidoreductase subunit 5 | - | 49 | - | - | 0 | 2238 |
| *rpl32* | ribosomal protein L32 | - | 3 | - | - | 0 | 156 |
| *ccsA* | cytochrome c heme attachment protein | - | 33 | - | - | 0 | 984 |
| *ndhD* | NADH-plastoquinone oxidoreductase subunit 4 | - | 30 | - | - | 0 | 1518 |
| *psaC* | photosystem I subunit VII | - | 1 | - | - | 0 | 243 |
| *ndhE* | NADH-plastoquinone oxidoreductase subunit 4L | - | 2 | - | - | 0 | 303 |
| *ndhG* | NADH-plastoquinone oxidoreductase subunit 6 | - | 11 | - | - | 0 | 528 |
| *ndhI* | NADH-plastoquinone oxidoreductase subunit I | - | 9 | - | - | 0 | 501 |
| *ndhA* | NADH-plastoquinone oxidoreductase subunit 1 | 37 | 17 | 13 | - | 1091 | 1080 |
| *ndhH* | NADH-plastoquinone oxidoreductase subunit 7 | - | 22 | - | - | 0 | 1179 |
| *rps15* | ribosomal protein S15 | - | 5 | - | - | 0 | 264 |
| *ycf1.2^a^* | hypothetical chloroplast RF19 | - | 237 | - | - | 0 | 5307 |

a The gene was located at IRb and had different variation number with the corresponding gene in IRa.
